# Acute and chronic mitochondrial respiratory chain deficiency differentially regulate lysosomal biogenesis

**DOI:** 10.1038/srep45076

**Published:** 2017-03-27

**Authors:** Lorena Fernández-Mosquera, Cátia V. Diogo, King Faisal Yambire, Gabriela L. Santos, Marta Luna Sánchez, Paule Bénit, Pierre Rustin, Luis Carlos Lopez, Ira Milosevic, Nuno Raimundo

**Affiliations:** 1 Institute of Cellular Biology, University Medical Center Goettingen, Goettingen, Germany; 2 Doctoral Program on Molecular Medicine, University of Goettingen, Goettingen, Germany; 3 International Max-Planck Research School on Neuroscience, Goettingen, Germany; 4 Departamento de Fisiología, Facultad de Medicina and Instituto de Biotecnología, Centro de Investigación Biomédica, University of Granada, Granada, Spain; 5 INSERM UMR 1141, Hôpital Robert Debré, Paris, France; 6 Faculté de Médecine Denis Diderot, Université Paris Diderot – Paris 7, Site Robert Debré, Paris, France; 7 European Neuroscience Institute, Goettingen, Germany

## Abstract

Mitochondria are key cellular signaling platforms, affecting fundamental processes such as cell proliferation, differentiation and death. However, it remains unclear how mitochondrial signaling affects other organelles, particularly lysosomes. Here, we demonstrate that mitochondrial respiratory chain (RC) impairments elicit a stress signaling pathway that regulates lysosomal biogenesis via the microphtalmia transcription factor family. Interestingly, the effect of mitochondrial stress over lysosomal biogenesis depends on the timeframe of the stress elicited: while RC inhibition with rotenone or uncoupling with CCCP initially triggers lysosomal biogenesis, the effect peaks after few hours and returns to baseline. Long-term RC inhibition by long-term treatment with rotenone, or patient mutations in fibroblasts and in a mouse model result in repression of lysosomal biogenesis. The induction of lysosomal biogenesis by short-term mitochondrial stress is dependent on TFEB and MITF, requires AMPK signaling and is independent of calcineurin signaling. These results reveal an integrated view of how mitochondrial signaling affects lysosomes, which is essential to fully comprehend the consequences of mitochondrial malfunction, particularly in the context of mitochondrial diseases.

Mitochondria are fundamental organelles in metabolism and cell biology^1^. In addition to the long characterized metabolic roles, including energy metabolism and citrate cycle, iron and calcium metabolism and regulation of apoptosis, mitochondria are increasingly recognized as key signaling platforms affecting not only major cellular processes but also far-reaching systemic regulatory mechanisms^2^. Furthermore, it is now clear that mitochondria interact with other organelles, both through physical contact sites and through signaling pathways^3^,^4^. The interaction between mitochondria and the endoplasmic reticulum (ER) provides pivotal examples of both: the mitochondrial-ER contact sites have been extensively studied and are known to be key players in cellular calcium homeostasis^5^, while mitochondrial dysfunction has also been shown to trigger ER stress^6^.

Recently, it was also reported that acute mitochondrial malfunction results in impairment of lysosomes and induction of lysosomal biogenesis^7^.

The lysosomes are part of the endolysosomal system, and have traditionally been associated with degradation of cellular components^8^,^9^. In the recent years, the lysosomes have been shown to be much more than an acid recycling bag, as their role in amino acid sensing and autophagy regulation has been elucidated^8^,^9^. Furthermore, a lysosomal stress response that increases lysosomal biogenesis was also identified^10^,^11^. This stress response is mediated by the transcription factors of the microphtalmia family, TFEB, MITF, TFEC and TFE3^10^,^11^,^12^. These transcription factors are associated to the lysosome during basal conditions, where they become phosphorylated by the mTORC1 complex. Upon lysosomal stress or amino acid starvation, mTORC1 is inactivated and Ca^2+^ is released from the lysosomes, resulting in the activation of calcineurin which can dephosphorylate TFEB and related molecules^13^. Dephosphorylated TFEB can then relocate to the nucleus and drive its transcriptional program. TFE3, TFEC and MITF seem to have similar regulatory mechanisms.

Under acute mitochondrial stress, lysosomes become dysfunctional and TFEB relocates to the nucleus^14^,^15^,^16^. The mechanisms linking these processes remain however not completely understood^7^. In this study, we show how acute and chronic respiratory chain defects have opposite effects on lysosomal biogenesis, both in cultured cells and *in vivo* in a mouse model of respiratory chain dysfunction. While acute mitochondrial stress triggers a AMPK-TFEB/MITF pathway that leads to increased lysosomal biogenesis, chronic mitochondrial stress results in repression of lysosomal biogenesis.

## Results

### Lysosomal biogenesis is differentially regulated by acute and chronic mitochondrial dysfunction

**Figure 1:**
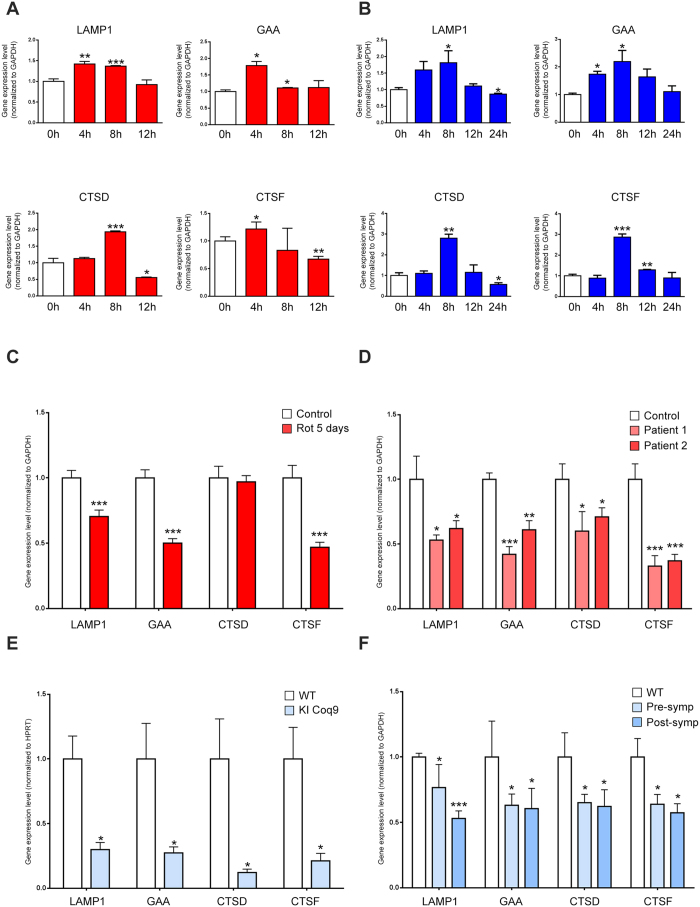
Acute and chronic mitochondrial malfunction differentially affect the transcriptional program of lysosomal biogenesis. (**A**) Relative transcript levels of LAMP1, GAA, CTSD and CTSF in MEFs treated with the respiratory chain complex I inhibitor rotenone (250 nM), collected at 0, 4, 8 and 12 hours of treatment, normalized to GAPDH. The bars represent average and standard error of the mean (S.E.M.) (N = 4) with p-values indicated (*p < 0.05; **p < 0.005; ***p < 0.001). (**B**) Relative transcript levels of LAMP1, GAA, CTSD and CTSF in MEFs treated with the uncoupler carbonyl cyanide 3-chlorophenylhydrazone (CCCP) (10 μM), collected at 0, 4, 8, 12 and 24 hours of treatment, normalized to GAPDH. The bars represent average and S.E.M. (N = 4) with p-values indicated (*p < 0.05; **p < 0.005; ***p < 0.001). (**C**) Relative transcript levels of LAMP1, GAA, CTSD and CTSF in MEFs treated with the respiratory chain complex I inhibitor rotenone (250 nM) for 5 days, normalized to GAPDH. The white bar corresponds to the control MEFs (treated with DMSO) and the red bars to the MEFs treated with rotenone. The bars represent average and S.E.M. (N = 4); t test p-values (*p < 0.05; **p < 0.005; ***p < 0.001). (**D**) Relative transcript levels of LAMP1, GAA, CTSD and CTSF in fibroblasts from patients with mutations in respiratory chain complex I subunits (red bars) and control individuals (white), normalized to GAPDH. Patient 1 presents a mutation in the NDUFV1 subunit^26^ and patient two has a mutation in the NDUFV2 subunit^27^. The bars represent average and S.E.M. (N = 4) with p-values indicated (*p < 0.05; **p < 0.005; ***p < 0.001). (**E**) Relative transcript levels of LAMP1, GAA, CTSD and CTSF in MEFs from Coq9^R239X^ mice, normalized to HPRT. The white bar corresponds to the wild-type mice MEFs and the blue bars to the Coq9^R239X^ MEFs. The bars represent average and S.E.M. (N = 4) with t test p values indicated (*p < 0.05; **p < 0.005; ***p < 0.001). (**F**) Relative transcript levels of LAMP1, GAA, CTSD and CTSF in heart from Coq9^R239X^ mice, normalized to GAPDH. The white bars corresponds to the wild-type mice, the blue bars to the Coq9^R239X^. The bars represent average and S.E.M. (N = 5) with p-values indicated (*p < 0.05; **p < 0.005; ***p < 0.001).

To assess how lysosomal biogenesis is affected by mitochondrial malfunction, we first subjected wild-type mouse embryonic fibroblasts (MEF) to a mitochondrial respiratory chain (RC) complex I inhibitor, rotenone (250 nM). We observed that the transcript levels of several lysosomal genes are rapidly increased upon RC complex I inhibition, and eventually return to baseline levels after 12 h treatment (Fig. 1A). This result suggests that the effect of mitochondrial malfunction on lysosomal biogenesis is dependent on the duration of the mitochondrial perturbation. We then performed a similar experiment, but treating the MEF with a mitochondrial uncoupler, carbonyl cyanide 3-chlorophenylhydrazone (CCCP, 10 μM), which is widely used to induce mitophagy. Upon CCCP treatment, the expression of lysosomal genes increases rapidly and returns to baseline, albeit later than the rotenone treatment (Fig. 1B). Overall, the pattern resulting from perturbation of mitochondrial respiratory chain and oxidative phosphorylation is the same: rapid up-regulation of lysosomal genes, followed by a return to the baseline after few hours. The longer curve observed in CCCP treatment may be related to the relative effect of the two treatments on mitochondrial inner membrane potential (ΔΨ_m_): rotenone has a small effect on ΔΨ_m_ while CCCP strongly affects ΔΨ_m_ (Supplementary Figure 1A). The effects of rotenone and CCCP on the overall activity of the respiratory chain, as measured by O_2_ consumption, show that the treatments are working as expected (Supplementary Figure 1B). Furthermore, these results show that the effect of acute and chronic mitochondrial respiratory chain deficiency in lysosomal biogenesis may be different. To test this, we treated MEFs with rotenone (250 nM) for 5 days, and observed a strong reduction in the transcript levels of three of the lysosomal genes (cathepsin D was not significantly changed) (Fig. 1C). We then tested other chronic models of respiratory chain deficiency, to further validate this result. First, we used patient fibroblasts with complex I deficiency. These cells are permanently deficient in complex I activity, and thus represent a model of chronic respiratory chain deficiency^17^,^18^. Accordingly, the transcript levels of the four tested lysosomal genes are significantly repressed in these cells (Fig. 1D). Then, we tested MEFs obtained from a mouse model of mitochondrial malfunction (Coq9^R239X^, knock-in of a patient mutation in Coq9). These mice lack a functional COQ9 protein and present instability of mitochondrial supercomplex I-III^19^. The transcript levels of the four lysosomal genes tested were robustly decreased in Coq9^R239X^ MEFs (Fig. 1E). Finally, we tested the heart of the Coq9^R239X^ mice, in which the transcript levels of the four lysosomal genes were significantly decreased (Fig. 1F). These results, obtained by long-term chemical inhibition of the respiratory chain and genetic impairment of the respiratory chain in fibroblasts, as well as by genetic impairment of the respiratory chain *in vivo*, support a model in which acute mitochondrial respiratory chain deficiency triggers lysosomal biogenesis, but chronic mitochondrial respiratory chain deficiency represses it.

**Figure 2:**
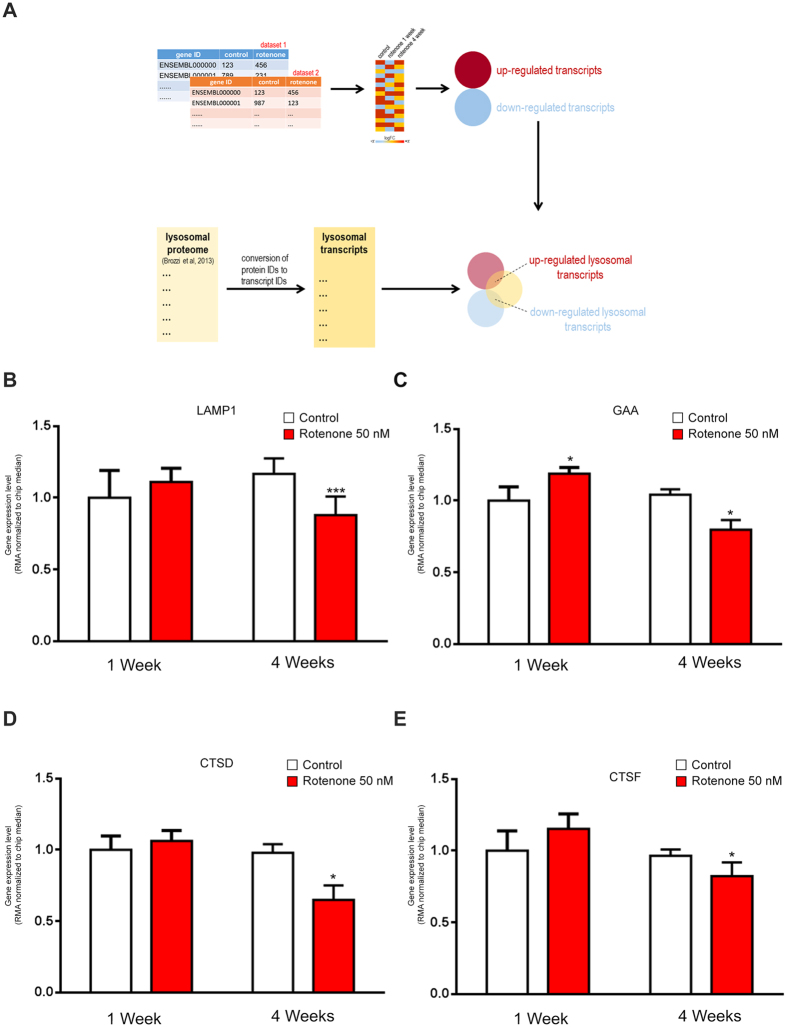
Effect of rotenone on lysosomal gene expression in SK-N-MC neuroblastoma cells. (**A**) Illustration of the experimental strategy employed to determine lysosomal-related genes that are significantly affected by inhibition of the mitochondrial respiratory chain. (**B**) Relative transcript levels of LAMP1 in SK-N-MC neuroblastoma cells treated with the respiratory chain complex I inhibitor rotenone (50 nM) collected at 1 and 4 weeks of treatment, after RMA normalization. The white bar corresponds to the control MEFs and the red bars to the MEFs treated with rotenone with t test p-values indicated (*p < 0.05; **p < 0.005; ***p < 0.001). (**C**) Relative transcript levels of GAA in SK-N-MC neuroblastoma cells treated with the respiratory chain complex I inhibitor rotenone (50 nM) collected at 1 and 4 weeks of treatment, after RMA normalization. The white bar corresponds to the control MEFs and the red bars to the MEFs treated with rotenone with t test p-values indicated (*p < 0.05; **p < 0.005; ***p < 0.001). (**D**) Relative transcript levels of CTSD in SK-N-MC neuroblastoma cells treated with the respiratory chain complex I inhibitor rotenone (50 nM) collected at 1 and 4 weeks of treatment, after RMA normalization. The white bar corresponds to the control MEFs and the red bars to the MEFs treated with rotenone with t test p-values indicated (*p < 0.05; **p < 0.005; ***p < 0.001). (**F**) Relative transcript levels of CTSF in SK-N-MC neuroblastoma cells treated with the respiratory chain complex I inhibitor rotenone (50 nM) collected at 1 and 4 weeks of treatment, after RMA normalization. The white bar corresponds to the control MEFs and the red bars to the MEFs treated with rotenone with t test p-values indicated (*p < 0.05; **p < 0.005; ***p < 0.001).

However, given that we observed a limited number of genes, we could not guarantee that the hundreds of genes that encode for lysosomal proteins have a similar behavior. Therefore, we took advantage of two publicly-available transcriptional datasets of dopaminergic neuroblastoma cells subject to rotenone treatment (GSE35642 and GSE4773). We used a database of lysosomal proteins^20^ to define a list of “lysosomal transcripts” (Supplementary Table 1). This “lysosomal transcripts” list allows us to study the overall effect of mitochondrial impairment over the expression of lysosomal genes, by monitoring how many of those genes had significantly changed expression upon rotenone treatment, and in which direction (up-regulated or down-regulated) – the experimental strategy is illustrated in Fig. 2A. In the first study^21^ (GSE35642), after one week of treatment with two different concentrations of rotenone (5 nM and 50 nM), there was a higher number of up-regulated lysosomal genes, suggesting an overall increase in lysosomal biogenesis (Supplementary Figure 2A). However, after four weeks of treatment the effect was completely reversed, with a much higher number of lysosomal genes being down-regulated (Supplementary Figure 2A). In the second study^22^ (GSE4773), the number of up- and down-regulated genes was similar after one week of treatment, but the amount of down-regulated genes increased progressively at 2- and 4-weeks of treatment (Supplementary Figure 2B).

We then tested how the four genes that we have used as proxy for lysosomal biogenesis were affected. We focused on the study which employed 50 nM rotenone treatment, which is closer to our conditions (GSE35642). None of the four genes was down-regulated after 1 week of 50 nM rotenone treatment, but all four genes tested showed a significant down-regulation after 4 weeks of treatment (Fig. 2B–E). These results are in agreement with the long-term effects of respiratory chain inhibition that we described in Fig. 1. Furthermore, they show that these four genes constitute a good proxy for the rest of the lysosomal genes.

We further used the GSE35642 dataset to determine how the genes that are changed after 1 week of 50 nM rotenone treatment behave at four weeks of rotenone treatment in neuroblastoma cells. We filtered the genes whose expression was significantly changed both at 1-week and 4-week treatments, and followed what happens to the direction of the change. Of the 32 transcripts meeting these criteria, 23 were increased after 1 week, and 9 decreased. Remarkably, 22 out of the 23 transcripts increased at 1 week were significantly down-regulated after 4 weeks of 50 nM rotenone treatment (Supplementary Figure 2C and Supplementary Table 2). Furthermore, the 9 transcripts that were down-regulated at week 1, were all up-regulated at week 4 (Supplementary Figure 2C, and Supplementary Table 2).

Altogether, these results show that lysosomal biogenesis undergoes a biphasic response to mitochondrial malfunction, with up-regulation of lysosomal biogenesis after the onset of mitochondrial respiratory chain deficiency, followed by repression after persistence of the mitochondrial defect.

### Induction of lysosomal biogenesis by acute mitochondrial stress is TFEB/MITF-dependent

**Figure 3:**
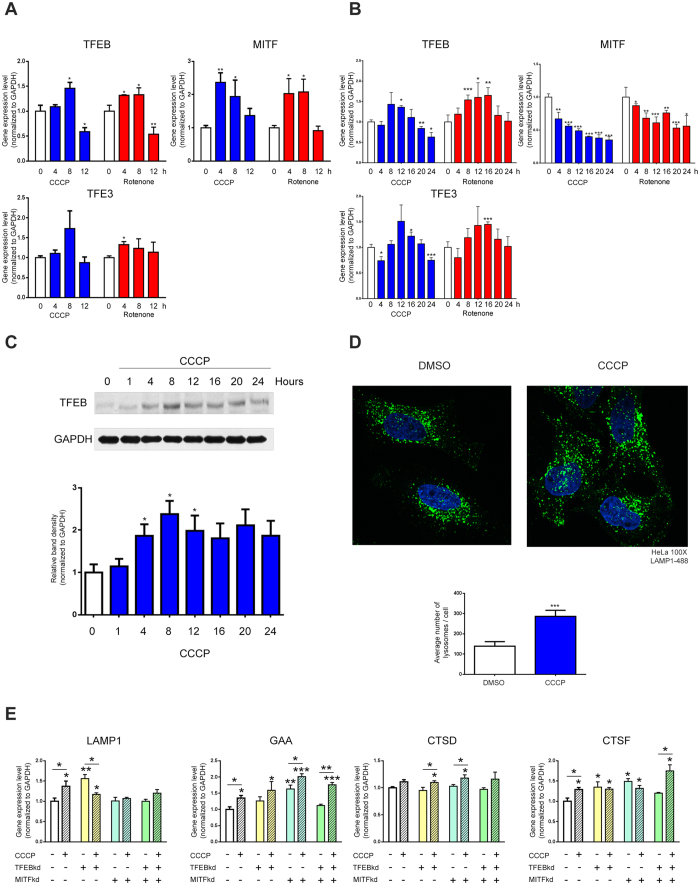
Acute mitochondrial stress triggers TFEB/MITF-dependent lysosomal biogenesis. (**A**) Relative transcript levels of TFEB, MITF and TFE3 in MEFs treated with DMSO (controls, white bars), rotenone (250 nM, red) and CCCP (10 μM, blue), collected at 0, 4, 8 and 12 hours of treatment, normalized to GAPDH mRNA transcript level. The bars represent average and S.E.M. an (N = 4) with p-values indicated (*p < 0.05; **p < 0.005; ***p < 0.001). (**B**) Relative transcript levels of TFEB, MITF and TFE3 in HeLa cells treated with DMSO (controls, white bars) rotenone (250 nM, red) and CCCP (10 μM, blue), collected at 0, 4, 8, 12, 16, 20 and 24 hours of treatment, normalized to GAPDH mRNA transcript level. The bars represent average and S.E.M. (N = 6) with p-values indicated (*p < 0.05; **p < 0.005; ***p < 0.001). (**C**) Western blot analysis of whole-cells extracts for TFEB in HeLa cells treated with CCCP (10 μM), collected at 0, 1, 4, 8, 12, 16, 20 and 24 hours of treatment, and corresponding quantification (normalized to GAPDH). The white bar corresponds to the control HeLa cells and the blue bars to the HeLa cells treated with CCCP. The bars represent average and S.E.M. (N > 4) with p-values indicated (*p < 0.05; **p < 0.005; ***p < 0.001). (**D**) Representative spinning-disk microscopy images of HeLa cells treated 4 hours with CCCP 10 μM. The cells were immunostained with LAMP1-488 and DAPI. The number of the lysosomes was quantified using ImageJ. The average number of lysosomes and respective standard error of the mean are represented in the graph below. At least 15 cells of each group were used for the quantification. The statistics was done using t test, and the p-value is indicated (*p < 0.05; **p < 0.005; ***p < 0.001). (**E**) Transcript levels of LAMP1, GAA, CTSD and CTSF mRNA transcript levels in TFEB/MITFkd HeLa cells treated 4 hours with DMSO or CCCP (10 μM) normalized to GAPDH. The bars represent average and S.E.M. (N = 4) with t test p-values indicated (*p < 0.05; **p < 0.005; ***p < 0.001).

Given the known role of TFEB in the regulation of a gene expression program of lysosomal biogenesis, we addressed the role of TFEB in the response to mitochondrial respiratory chain deficiency. We first observed that the transcript levels of TFEB and other related microphtalmia transcription factors (MITF, TFE3) were following a pattern very similar to the lysosomal transcripts: rapid up-regulation in response to mitochondrial stress followed by return to the baseline, both under rotenone or CCCP treatment (Fig. 3A). The transcript levels of TFEC, another microphtalmia transcription factor, were very low in MEF and its quantification wasn’t sufficiently reliable. To directly assess if TFEB was involved in the lysosomal biogenesis induced by acute mitochondrial stress, we decided to move to another cell type more amenable to genetic manipulation than primary MEF. We employed HeLa cells, which are easier to transfect and not limited by early passage number. We first verified that the treatment of HeLa cells with rotenone and CCCP affects TFEB in a manner similar to what we observed in MEF, which was the case (Fig. 3B). The levels of TFEB protein follow a similar pattern (Fig. 3C). Accordingly, a 4-hour treatment of HeLa cells with CCCP results in an increase in the number of lysosomes (Fig. 3D).

We then prepared a stable knock-down of TFEB in HeLa cells, and verified the efficiency of the silencing by western blotting (Supplementary Figure 3A). We tested five different constructs, independently, and picked the two clones with the most efficient silencing for further studies. Since MITF also has the ability to trigger lysosomal biogenesis, we decided to also knock-down MITF both independently and together with TFEB knock-down. We used siRNA against MITF in the scrambled and stable TFEB knock-down lines. We assessed the efficiency of MITF knock-down by qPCR (Supplementary Figure 3B), and also verified that the knock-down of MITF does not affect TFEB levels and vice-versa (Supplementary Figure 3B). We then treated the scrambled, TFEB knock-down, MITF knock-down and double TFEB/MITF knock-down with CCCP and determined how the transcript levels of lysosomal genes are affected. As expected, CCCP treatment increased the levels of LAMP1 and GAA in scrambled cells, but was unable to do so in TFEB knock-down and double TFEB/MITF knock-down cells (Fig. 3E). MITF knock-down was sufficient to inhibit the induction of LAMP1 but not of GAA (Fig. 3E). These results show that lysosomal biogenesis induced by acute mitochondrial stress is TFEB/MITF dependent.

### AMPK signaling is necessary for lysosomal biogenesis induced by acute mitochondrial stress

**Figure 4:**
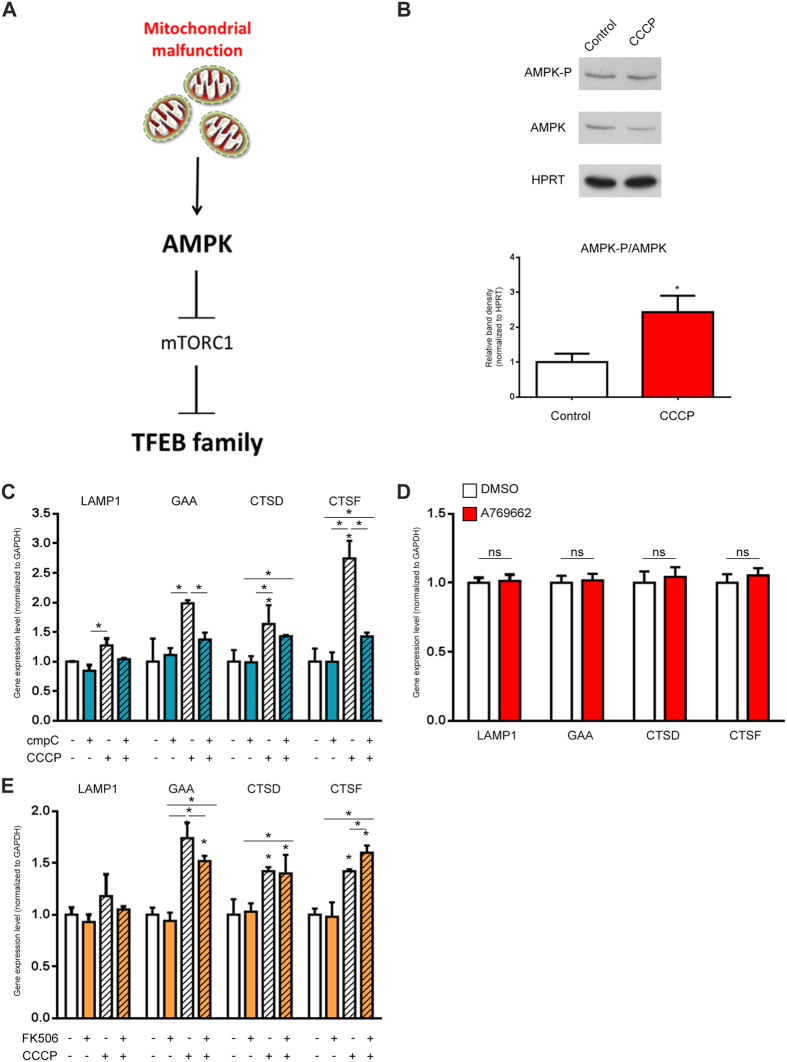
Lysosomal biogenesis triggered by acute mitochondrial stress requires AMPK signaling. (**A**) Mitochondrial stress leads in lysosomal biogenesis through TFEB. The damage mitochondria release signalling that affects AMPK, which is involved in lysosomal biogenesis through TFEB family pathway. (**B**) Western blot analysis of whole-cells extracts showing increased AMPK activation, by measuring AMPK and AMPK-P in HeLa cells treated 4 hours with CCCP (10 μM). The graph shows the ratio of AMPK-P/AMPK protein normalized to HPRT. The white bar corresponds to the control HeLa cells and the red bar to the HeLa cells treated with CCCP. The bars represent average and S.E.M. (N = 4) with t test p-value indicated (*p < 0.05; **p < 0.005; ***p < 0.001). (**C**) Transcript levels of LAMP1, GAA, CTSD and CTSF mRNA transcript levels in HeLa cells treated 4 hours with CCCP 10 μM and dorsomorphin (compound C) (10 μM), normalized to GAPDH mRNA transcript level. The white bar corresponds to the control HeLa cells, the bars with pattern to HeLa cells treated with CCCP and the blue bars corresponds to HeLa cells treated with compound C. The bars represent average and S.E.M. (N = 6) with p-values indicated (*p < 0.05; **p < 0.005; ***p < 0.001). (**D**) Transcript levels of of LAMP1, GAA, CTSD and CTSF mRNA transcript levels in HeLa cells treated 4 hours with A769962, normalized to GAPDH mRNA transcript level. The white bar corresponds to the control HeLa cells and the red bars to the HeLa cells treated with A769662 (100 μM). The bars represent average and S.E.M. (N = 4). No statistically significant changes were observed. (**E**) Transcript levels of LAMP1, GAA, CTSD and CTSF mRNA transcript levels in HeLa cells treated 4 hours with FK506 (5 μM) and CCCP (10 μM), normalized to GAPDH mRNA transcript level. The white bar corresponds to the control HeLa cells, the bars with pattern to HeLa cells treated with CCCP and the orange bars corresponds to HeLa cells treated with FK506. The bars represent average and S.E.M. (N = 6) with p-values indicated (*p < 0.05; **p < 0.005; ***p < 0.001).

We then set to determine what are the mechanisms linking acute mitochondrial stress with activation of TFEB/MITF signaling. We reasoned that given the known role of AMPK as a major responder to mitochondrial stress, and an inhibitor of mTORC1, which in turn inhibits TFEB activity, AMPK might be a key mediator between mitochondrial stress and TFEB activity (illustrated in Fig. 4A). Accordingly, AMPK phosphorylation is increased after 4 h treatment with 10 μM CCCP (Fig. 4B). Thus, we subjected HeLa cells to a 4-hour 10 μM CCCP treatment and simultaneously treated one group of cells with dorsomorphine (also referred to as “compound c”), a commonly used inhibitor of AMPK^23^. The treatment with CCCP expectedly increased the transcript levels of lysosomal genes LAMP1, GAA, CTSD and CTSF, but this increase was blocked or strongly ablated in the presence of dorsomorphin (Fig. 4C). These results show that AMPK signaling is needed for the activation of TFEB by acute mitochondrial stress. We next tested if activation of AMPK, in the absence of mitochondrial stress, was enough to trigger TFEB-dependent transcription. For this we treated HeLa cells with A769662, a commonly used AMPK activator^24^, for 4-hours and determined the transcript levels of lysosomal genes. No significant increase was observed (Fig. 4D), despite A769662 was able to activate AMPK (Supplementary Figure 4). This implies that AMPK activation *per se* is not sufficient to trigger TFEB-dependent transcription in the absence of mitochondrial stress.

In addition to the AMPK-mTORC1 axis, TFEB activity was recently shown to be also regulated by calcineurin. To assess the role of calcineurin in our experimental paradigm, we treated cells simultaneously with 10 μM CCCP and calcineurin inhibitor^13^ FK506, and found no effect on the transcript levels of TFEB targets (Fig. 4E). This result implies that the regulation of TFEB in response to acute mitochondrial stress occurs mostly through the AMPK-mTORC1 axis and is independent of calcineurin.

## Discussion

This study addresses the mechanisms by which mitochondrial malfunction, and particularly respiratory chain deficiency, impact lysosomal biogenesis. We show here that acute and chronic perturbations of the mitochondrial respiratory chain have opposite effects in lysosomal biogenesis: while acute mitochondrial respiratory chain stress triggers AMPK- and TFEB/MITF-dependent lysosomal biogenesis, chronic mitochondrial respiratory chain dysfunction actually results in a repression of TFEB/MITF transcriptional activity and lysosomal biogenesis.

The impact of mitochondrial defects in cellular function has been addressed by numerous studies in recent years. Several mechanisms of mitochondrial stress were identified, which culminate in the modulation of cellular signaling pathways and of gene expression programs^2^. The impact of mitochondrial defects in other organelles has been subject to less attention. Nevertheless, it was shown that mitochondrial malfunction can trigger endoplasmic reticulum stress and the unfolded protein response^6^. Recently, three studies addressed the effect of mitochondrial malfunction and of mitophagy on lysosomal function and biogenesis. In cultured cells, short-term mitophagy bursts affect the regulation of the microphtalmia transcription factor family^15^,^16^, which includes TFEB, MITF, TFEC and TFE3, while mtDNA depletion, and consequent loss of respiratory chain function, perturbs lysosomal function and triggers a program of incomplete lysosomal biogenesis in mouse T cells upon their activation^14^. However, the mechanisms underlying the impact of mitochondrial malfunction on lysosomes and lysosomal biogenesis remain unaddressed^7^. Here, we show that mitochondrial malfunction affects lysosomal biogenesis in a biphasic manner. Under acute mitochondrial stress, an AMPK-dependent activation of TFEB/MITF transcriptional response results in the induction of a transcriptional program of lysosomal biogenesis. This response fades after few hours of mitochondrial stress, and returns to basal levels. This biphasic effect is independent of the cell type, since we observed it in mouse embryonic fibroblasts and HeLa cells, and in transcriptional data from neuroblastoma cells. Rather, this biphasic effect seems to be a common response to different mitochondrial stresses employed in our study, such as inhibition of respiratory chain or uncoupling the respiratory chain from the oxidative phosphorylation machinery. Interestingly, in chronic models of mitochondrial malfunction, we find the transcriptional program of lysosomal biogenesis to be repressed. This is observed *in vivo* in a mouse model of mitochondrial respiratory chain deficiency, in the MEFs obtained from the same mouse, as well as in patient fibroblasts with complex I deficiency. Therefore, the effect is valid in post-mitotic tissues as well as primary cells in culture. Our model of lysosomal biogenesis activation by acute mitochondrial stress is in agreement with other models of acute mitochondrial stress, such as mitophagy bursts and T-cell activation, which also induce TFEB and lysosomal biogenesis^14^,^15^,^16^. The exact kinetics of the biphasic effect may vary depending on the cell type employed, the treatment (compound, concentration) and the genetic environment. For example, Ivankovic *et al*. show that TFEB mRNA peaks after 18 hours 10 μM CCCP treatment of SH-SY5Y neuroblastoma cells^15^. In our MEFs and HeLa the same concentration of CCCP induces TFEB much earlier, and by that time point TFEB is already down. It is possible that differences regarding the metabolic functions of MEF and neuroblastoma cells contribute to the different kinetics of the biphasic effect. The 250 nM rotenone treatment in our MEFs also seems to have faster kinetics than a 50 nM rotenone treatment in neuroblastoma cells^21^,^22^, but in this case the increased rotenone concentration is likely a factor. Nevertheless, the rotenone treatments in neuroblastoma cells concur with our results in that lysosomal genes eventually become repressed after a long-term treatment. This is in agreement with our long-term treatment with rotenone, but also with the MEFs and tissues from Coq9 mice, and patient fibroblasts with deficiency in complex I, all of which are models of chronic mitochondrial respiratory chain deficiency, and all of which show a robust down-regulation of the lysosomal genes that we were monitoring. The study by Baixauli *et al*. shows that in cytoplasmic hybrid L929 mouse cells with defects in complex I subunit ND6, TFEB transcript levels are up but LAMP1 transcript levels are not changed^14^. These results show a different pattern than what we observe in chronic mitochondrial malfunction. It is unlikely that the difference is due to different control genes in the qPCR reactions (beta-actin and beta-microglobulin in Baixauli and colleagues, HPRT and GAPDH in this manuscript), since when we use beta-actin as a control gene we observe the same results as with GAPDH and HPRT (data not shown). It is possible that the long ethidium bromide treatment that the original L929 cells underwent to completely lose their mtDNA has impacted the TFEB signaling pathway – certainly the near future will shed light on how different cell types and different stresses impact how mitochondria and lysosomes communicate. Our result showing down-regulation of lysosomal genes upon long-term mitochondrial stress is consistent across several models (rotenone-treated MEFs, patient fibroblasts, MEFs with genetic inhibition of the RC, tissues from mice with mitochondrial RC defects).

The initial increase in lysosomal biogenesis in response to mitochondrial stress is likely to allow the cells to cope with the increase in autophagy flux and to increase the capacity to remove damaged mitochondria. At some point, after about 12–24 h of mitochondrial stress, the cells start deactivating the transcriptional mechanisms leading to the generation of more lysosomal proteins, possibly as a feed-back mechanism designed to avoid excessive accumulation of lysosomes in the cells. The lysosomes are involved not only in the autophagy pathway but also in endocytic pathway and in exocytosis of cellular components, and thus accumulation of lysosomes might have detrimental effects to other cellular functions^25^. The importance of avoiding excessive lysosomal biogenesis is underscored by the repression of the transcript levels of lysosomal genes under chronic mitochondrial stress. Interestingly, mitochondrial malfunction seems to be associated with lysosomal impairments, both in acute (TFAM−/− T cells upon activation)^14^ and chronic stress (e.g., OPA1−/−, AIF−/− MEFs)^26^, characterized by enlarged dysfunctional lysosomes akin to the saturated organelles observed in lysosomal storage diseases.

Interestingly, AMPK signaling is needed to activate lysosomal biogenesis upon acute mitochondrial malfunction. AMPK is a known responder to mitochondrial stress^2^, and while the exact mechanism leading to AMPK activation in acute mitochondrial stress remains to be determined, it is likely that several of its known activators, such as ROS^23^, Ca^2+^ and lower energy charge^27^,^28^, are signals of acute mitochondrial stress. AMPK has broad roles in cellular signaling^28^, namely in the regulation of compensatory mitochondrial biogenesis^27^, activation of autophagy and the regulation of mTORC1 activity^28^. Furthermore, AMPK was recently shown to have the ability to regulate TFEB activity in embryonic stem cells^24^. The known roles of AMPK and its requirement for lysosomal biogenesis upon acute mitochondrial stress suggest a coordinated cellular response: the increase in AMPK signaling will likely result in ULK1/2 activation and increased formation of autophagosomes^29^, which can then be used to remove damaged mitochondria from the cytoplasm. However, such a burst of autophagosomes might saturate the lysosomal capacity, and thus the simultaneous induction of lysosomal biogenesis allows for the generation of new lysosomes, enabling them to cope with the increased autophagic flow.

The regulation of TFEB under acute mitochondrial stress seems to be independent of calcineurin. The mechanisms regulating TFEB activity only recently started to be identified, and additional pathways have been proposed that are independent of the initially described roles of mTORC1 and calcineurin^30^. As already suggested by Nezich and colleagues, the regulation of TFEB intracellular localization and activity seems more complex than initially thought and requires further investigation^16^. Our data on the role of TFEB in response to acute mitochondrial stress is in agreement with the works by Nezich and colleagues^16^ as well as Ivankovic and colleagues^15^, which both demonstrate that TFEB and its related transcription factors are necessary for the increase in lysosomal biogenesis upon mitophagy.

This study unveils a mechanism for the communication between mitochondria and lysosomes under acute mitochondrial stress, and highlights the importance of distinguishing between acute and chronic mitochondrial stresses and the respective cellular responses, in particular regarding the impact in other organelles. While this closely concerns mitochondrial diseases, the impact of dysfunctional mitochondria on other organelles is likely to be relevant for the understanding of neurodegenerative syndromes that involve chronically defective mitochondria (e.g., Parkinson’s disease triggered by mutations in mitochondrial genes such as POLG and PARK2, as well as rotenone-induced Parkisonism).

## Methods

### Drugs and cellular treatments

The following drugs were used: Carbonyl cyanide 3-chlorophenylhydrazone (CCCP) 10 μM (Fluka), Rotenone 250 nM (Sigma-Aldrich), dorsomorphin (compound C) 10 μM (InvivoGen), A769662 100 μM (Invivogen), FK506 (Tacrolimus) 5 μM (InvivoGen). The experiments were performed in the University Medical Center Göttingen according to the appropriate guidelines.

### Cell culture, stable and transient transfection

HeLa cells were grown in DMEM high glucose medium (Gibco) supplemented with 10% fetal bovine serum and 1% Penicillin/Streptomycin at 37 °C and 5% CO_2_, unless otherwise stated. Patient fibroblasts were collected and maintained according to the ethical guidelines of the Hospital Robert Depré Paris. RNA from patient fibroblasts was extracted using a Qiagen kit according to manufacturer instructions.

### Western Blotting

Whole-cell extracts of cultured human cells were prepared in 1,5% n-dodecylmaltoside in PBS as described (Raimundo *et al*., 2009). Gels were loaded with 50 μg of total protein per well, proteins were separated in 12% SDS-PAGE gels and transferred to polyvinylidene fluoride (PVDF) membranes (Amersham, Life Technologies). The following antibodies were used for immunoblotting: GAPDH (Sigma-Aldrich, G9545), HPRT (Abcam, ab10479), LAMP1 (Abcam, ab24170), TFEB (Novus, NBP1-67872), AMPKα1 (Cell Signaling, 2795), AMPKα1-P (T172) (Cell Signaling, 2535). Band density traces and quantification were determined using ImageJ.

### Quantitative RT-PCR

RNA extraction and purification were performed using Crystal RNA mini Kit (Biolab). RNA quantification and quality control were done using Nanodrop (PeqLab) and cDNA was synthesized with High-Capacity cDNA Reverse Transcription Kit (Applied Biosystems) according to the manufacturer’s instructions. cDNA was diluted 1:100, and each 8 μl reaction contained 4 μl diluted cDNA, 0,2 μl dilutions of each primer (from 25 μM stock), and 3,6 μl iTaq Universal SYBR Green Supermix (Bio-Rad).

### Lysosomal transcript list and data analysis

Using the Human Lysosome Gene Database (http://lysosome.unipg.it/)16, we defined a list of 435 genes encoding proteins with confirmed lysosomal localization. Then, using the gene symbol, we translated that list using the matrix file of the Affymetrix HG U133 Plus 2.0 microarray platform, which was used in the studies GSE35642 and GSE4773 were conducted. Of the 435 lysosomal genes, 418 had at least one probeset in HG U133 Plus 2.0. The list of these 418 genes, with their gene symbol and the AffyID is presented in Supplementary Table 1 (“lysosomal transcripts”). To determine how many of these genes had significant changes in expression upon rotenone treatments, we downloaded the CEL files of the GSE35642 and GSE4773 datasets. Each experiment was analyzed individually. For both, we normalized the samples by RMA and obtained a differentially-expressed gene (DEG) list as described^19^. Then, we determined how many of the “lysosomal transcripts” were included in the DEG for each condition. The result is presented in Fig. 2A and B.

### Flow Cytometry

Measurement of mitochondrial membrane potential with JC-1 20 μM (ThermoFisher Scientific) was performed according to the manufacturer instructions.

### Microscopy

Cells were plated using coverslips on 12 well-plates, treated with CCCP 10 μM (Fluka) and fixed with 4% PFA. The immunostaining was performed using the primary antibody H4A3 (DSHB) according to the manufacturer instructions.

### Mice Coq9^R239X^ sample preparation

RNA was extracted from heart of 3- (pre-symptomatic) and 5-month old (post-symptomatic) Coq9^R239X^ mice (C57BL/6 genetic background). Animal experiments were performed according to a protocol approved by the Institutional Animal Care and Use Committee of the University of Granada (procedures 62-CEEA-OH-2014) and were in accordance with the Directive 2010/63/EU on the protection of animals used for scientific purposes and the Spanish law (R.D. 53/2013).

### TFEB/MITF silencing

The HeLa cells with stable TFEB knock-down were prepared using lentiviral shRNA delivery. The shRNAs were purchased from Open Biosystems (TermoFisher Scientific). Lentiviral generation was done by growing HEK293T packaging cells in DMEM high glucose (Gibco) supplemented with 10% FBS and after 24 h were transfected with shRNA against target genes and viral components using Lipofectamine 2000, grown and concentrated using Lenti-X Concentrator (Clontech) according to the manufacturer’s instructions. HeLa cells were then seeded at 0.12 × 105 and grown overnight to 70–80% confluence. These cells were then transfected with the lentiviral particles using Polybrene (8 ug/ul). Puromycin (6 mg/m)-resistant transfected were selected as positive for knockdown of interest.

Transient MITF knockdowns were done using siRNAs and Fugene 6 Transfection Reagent (Promega) according to the manufacturer’s instructions. MITF knockdowns were done with RNAi Duplex Oligos from TriFECTa RNAi Kit (IDT) according to the manufacturer’s instructions.

### Statistical Analysis

For Western blotting, flow cytometry, quantitative RT-PCR, all data points represent the mean of at least three independent biological replicates, unless otherwise indicated in the respective figures. Error bars represent SEM. P-values were determined using Student’s t test for two group comparisons or ANOVA for multi-group comparisons. *p < 0.05 **p < 0.005 ***p < 0.001.

### Primers for qPCR

The primers used for the qPCR reactions are detailed in Supplementary Table 3.

## Additional Information

**How to cite this article:** Fernández-Mosquera, L. *et al*. Acute and chronic mitochondrial respiratory chain deficiency differentially regulate lysosomal biogenesis. *Sci. Rep.* **7**, 45076; doi: 10.1038/srep45076 (2017).

**Publisher's note:** Springer Nature remains neutral with regard to jurisdictional claims in published maps and institutional affiliations.

## Supplementary Material

Supplementary Figures

Supplementary Table 1

Supplementary Table 2

Supplementary Table 3
