# Supplementary material for: Acute and chronic mitochondrial respiratory chain deficiency differentially regulate lysosomal biogenesis
Source: Sci Rep. 2017 Mar 27;7:45076. doi: 10.1038/srep45076 (PMC5366864; doi:10.1038/srep45076)

# Supplementary Information

**Acute and chronic mitochondrial respiratory chain deficiency differentially regulate lysosomal biogenesis**

Lorena Fernández-Mosquera[1,2], Cátia V. Diogo[1], King F. Yambire[1,3], Gabriela L. Santos[1], Marta Luna Sánchez[4], Paule Bénit[5,6], Pierre Rustin[5,6], Luis Carlos Lopez[4], Ira Milosevic[7], Nuno Raimundo[1]*

**Supplementary Figure 1. Mitochondrial membrane potential and oxygen consumption.** **(A)** HeLa cells treated with rotenone (250 nM) and CCCP (10 µM) were incubated with JC-1 and the signal was measured by FACS. The white bar corresponds to the control HeLa cells, the red bar to the HeLa cells treated with rotenone and the blue bar to HeLa cells treated with CCCP. The ratio fluorescence is presented in the graph (average±standard deviation, N=3) with p-values indicated (*p < 0.05; **p < 0.005; ***p < 0.001).

**(B)** Mitochondrial O2 consumption rates of HeLa cells treated with rotenone (250 nM) and CCCP (10 µM). The white bar corresponds to the control HeLa cells, the red bar to the HeLa cells treated with rotenone and the blue bar to HeLa cells treated with CCCP. The ratio pmol/min/mg prot is presented in the graph (average±standard deviation, N=3) with p-values indicated (*p < 0.05; **p < 0.005; ***p < 0.001).

**Supplementary Figure 2. Effect of rotenone on lysosomal gene expression in SK-N-MC neuroblastoma cells.**

**(A-B)** The graphs represent the number of lysosomal genes whose expression is significantly changed by 1-4 week treatment with rotenone.

**(C)** Lysosomal genes whose expression is significantly changed at both 1 week and 4 weeks treatment with rotenone. The transcripts that are increased at 1 week are almost all significantly repressed at 4 weeks. The blue bar corresponds to 1 week of treatment and the red bar to 4 weeks of treatment. The logarithm of the fold change is represented.

**Supplementary Figure 3. Stable TFEB knock-down and transient MITF knock-down.**

**(A)** Five independent shRNAs were used to knock-down TFEB. The shRNAs were delivered via lentivirus. After selection, the cells were harvested to determine the efficiency of TFEB silencing by each construct.

**(B)** MITF was knocked-down using siRNAs. The efficiency of the knock-downs was assessed by quantitative PCR against a control gene. The transcript levels of TFEB were also measured. The values represented in the graph are the average ± standard deviation (N=3).

**Supplementary Figure 4. Effects of A769662 in AMPK.**

**(A)** Western blot analysis of whole-cells extracts for AMPK and AMPK-P in HeLa cells treated 4 hours with A769662 (100 µM). The graph shows the ratio of AMPK-P/AMPK protein normalized to HPRT. The white bar corresponds to the control HeLa cells and the

red bar to the HeLa cells treated with A769662. The bars represent average and standard error of the mean (N=4) with t test p values indicated (*p < 0.05; **p < 0.005; ***p < 0.001).

**Supplementary Table 1. Lysosomal transcript list.**

**Supplementary Table 2. Lysosomal transcripts changed after 1 week and 4 week treatment with 50nM rotenone in SH-SY5Y neuroblastoma cells.**

## Supplementary Figure 1

**A**

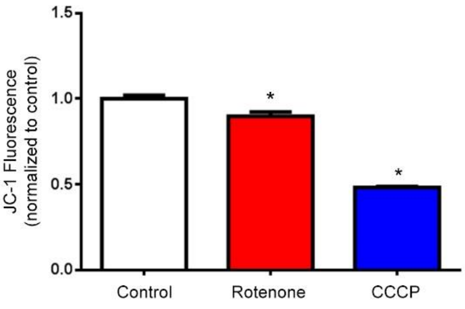


**B**

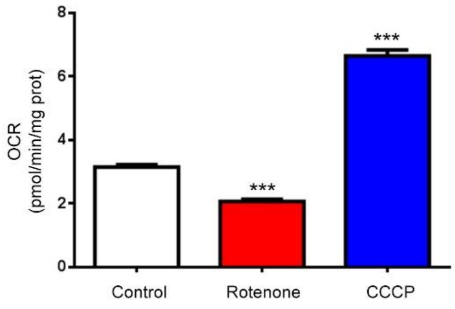

## Supplementary Figure 2

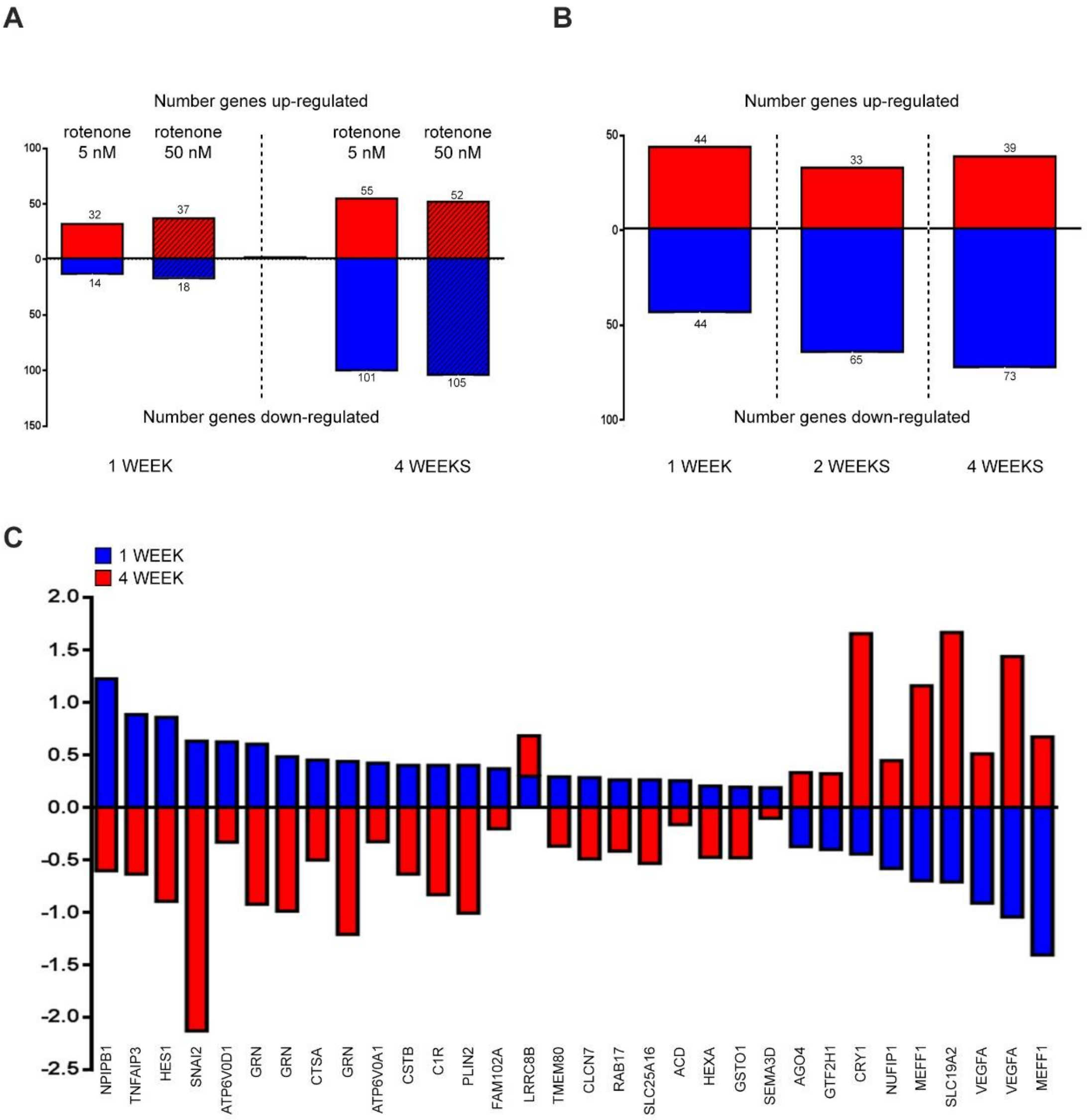

## Supplementary Figure 3

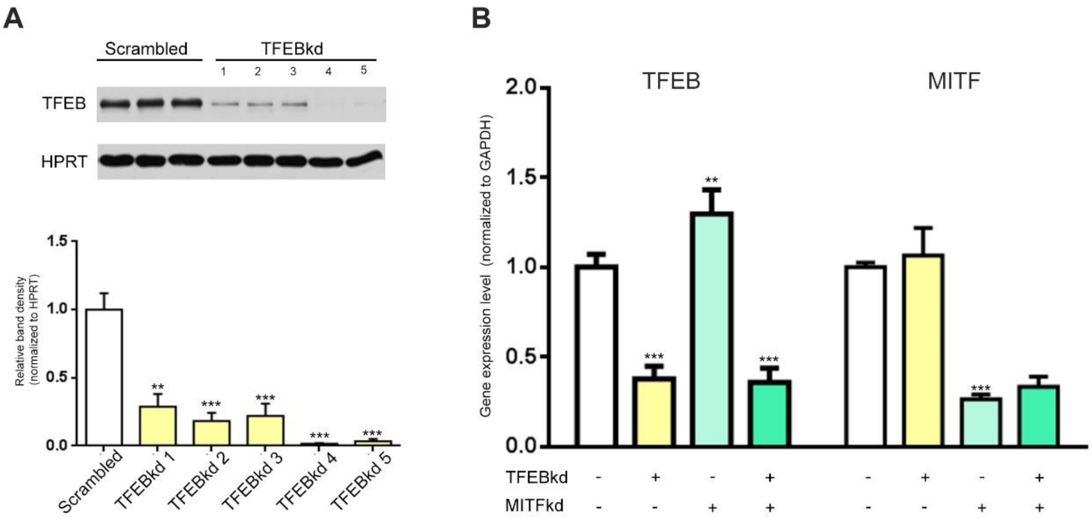

## Supplementary Figure 4

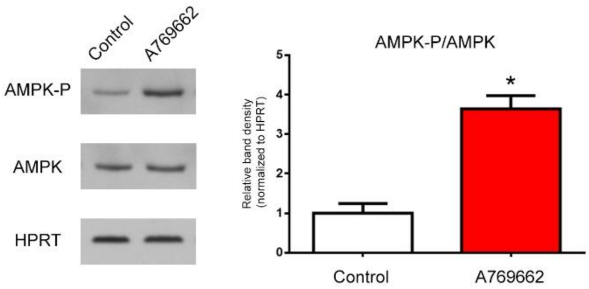

Supplement: Supplementary Figures [file srep45076-s1.pdf]
